# Challenges in Identifying Individualized Brain Biomarkers of Late Life Depression

**DOI:** 10.20900/agmr20230010

**Published:** 2024-01-24

**Authors:** Kayla Hannon, Janine Bijsterbosch

**Affiliations:** Department of Radiology, Washington University in St Louis, St Louis MO, 63110, USA

**Keywords:** late life depression, neuroimaging, biomarkers, personalized psychiatry

## Abstract

Research into neuroimaging biomarkers for Late Life Depression (LLD) has identified neural correlates of LLD including increased white matter hyperintensities and reduced hippocampal volume. However, studies into neuroimaging biomarkers for LLD largely fail to converge. This lack of replicability is potentially due to challenges linked to construct variability, etiological heterogeneity, and experimental rigor. We discuss suggestions to help address these challenges, including improved construct standardization, increased sample sizes, multimodal approaches to parse heterogeneity, and the use of individualized analytical models.

## INTRODUCTION

Late Life Depression (LLD) is defined as depression in older age. The age threshold is typically 60 years old but can range from 50 to 70 [1]. Depression is highly prevalent in older age; the prevalence of LLD was estimated to be 13.3% globally [2]. LLD is associated with cognitive deficits at a higher rate than depression experienced at a younger age [3–5]. Wang et al. 2022 found 26.6% of LLD patients showed significant cognitive impairment compared to healthy controls in all cognitive domains in the Mini-International Neuropsychiatric Interview [6]. LLD patients are also more likely to develop dementia [7] and Alzheimer’s [8], although the relationship between LLD and these conditions is still unclear [9]. Beyond associations with cognitive decline, depression in the older-aged population is associated with increased all-cause mortality risk [10–12]. Here, LLD has been associated with the increase and worsening of several conditions including frailty [13] and comorbidities like stroke [14], cardiovascular disease [15], heart failure [16], and cerebrovascular disease [17]. Late life depression is under diagnosed [18,19] and therefore often goes untreated, which indicates LLD as a potentially modifiable risk factor for a range of age-related pathologies. The goal of this review is to summarize the landmark historical and recent research investigating neuroimaging biomarkers in Late Life Depression, and to discuss the challenges the field faces in the search for replicable and generalizable biomarkers.

## NEURAL CORRELATES OF LATE LIFE DEPRESSION

Substantial work has investigated the neuroimaging correlates of LLD. Understanding the brain biomarkers of LLD may lead to a foundational understanding of the neurobiological mechanism of LLD that may impact clinical care, for example by developing diagnostic or treatment recommendations based on individuals’ MRI scans. As summarized below, LLD has been linked to changes in white matter (such as fractional anisotropy and white matter hyperintensities), reductions in gray matter volumes, and both hyper- and hypo-connectivity in functional connectivity networks. This section represents a critical review and overview of the existing literature, with an emphasis on meta-analyses. For full systematic reviews on neural correlates of Late Life Depression, we refer the reader to Wang et al. [20], Herrmann et al. [21], Sexton et al. [22], Wen et al. [23], Amidfar et al. [24], and Geerlings et al. [25].

### White Matter Abnormalities

LLD is most commonly associated with increased white matter hyperintensities [20], as they occur in this group at a higher rate than depression in earlier life [21]. White matter hyperintensities are white matter abnormalities, commonly lesions, that appear as hyperintensities on fluid attenuated inversion recovery MRI [26]. The association between LLD and increased white matter hyperintensities has been shown a number of times, including in systematic reviews [21,22] and a meta-analysis [22]. Furthermore, loss of white matter integrity as measured with diffusion weighted imaging (DWI) is also an important feature of LLD. A 2023 systematic review of 18 DWI studies found widespread white matter abnormalities in LLD, particularly reduced white matter integrity associated with cognitive impairment [9]. White matter integrity is commonly measured through fractional anisotropy, which estimates directional water flow in white matter axons (an indication of myelination) [27]. Sexton et al. 2011 [28] found LLD individuals performed significantly worse in several cognitive domains such as executive function and episodic memory. Reduced fractional anisotropy of the uncincate fasciculus was associated with reduced executive function, and episodic memory deficits were associated with reduced integrity of the corpus callosum. A 2014 meta-analysis on DWI studies found that fractional anisotropy in the dorsolateral prefrontal cortex and uncinate fasciculus was reduced in LLD, yet the study did not find consistent LLD-related fractional anisotropy changes in the corpus callosum or cingulum [23].

### Gray Matter Correlates

Several cortical and subcortical gray matter brain correlates of LLD have been identified. While reduced hippocampal volume is a robust finding in Major Depressive Disorder regardless of age [24,29], further reduction in hippocampal volume is a characteristic of LLD that has been well established [25,30–32]. Particularly, a meta-analysis [25] on 35 studies (2702 patients and 11,165 controls) found an overall effect of reduced hippocampal volume in LLD. Other subcortical regions have also been implicated in LLD, such as the amygdala [33,34], caudate [17,33,35], pallidum [33], putamen [17,33], and thalamus [17,33,36]. A meta-analysis in 2013 however only found significant reduction in gray matter volume in the hippocampus, putamen, and thalamus [22]. Frontal regions like the orbitofrontal cortex [33,37] and anterior cingulate cortex [33,38] have also commonly been noted to be smaller in LLD. However, the same meta-analysis only found the orbitofrontal cortex to have a significant reduction in gray matter volume [22].

### Functional Connectivity Correlates

Functional connectivity studies have identified several networks that are implicated in LLD, out of which the default mode network is the most well studied [39–41]. However, the direction of effect is unclear, as connectivity with the default mode network has been shown to be decreased [39,42] and increased [41,43] in LLD patients. Other networks of investigation in relation to LLD include the frontoparietal/central executive network [44], somatomotor network [39,44], auditory network [41], and visual network [41]. The salience network’s connectivity to the default mode network has been shown to be dysregulated in LLD. Andreescu et al. 2013 [45] investigated 47 older depressed patients and found increased posterior cingulate cortex (PCC)-prefrontal functional connectivity in treatment responsive individuals and increased PCC-striatum connectivity in treatment resistant individuals. The functional connectivity abnormalities went away once accounting for white matter hyperintensities however, indicating a mutual relationship between functional connectivity and white matter hyperintensities.

## SUBCLASSIFICATIONS OF LLD

Within LLD, there are subclassifications that may have important differences for the search for LLD brain biomarkers (Table 1). For example, late onset depression (LOD) is believed to have a different etiology than early onset depression (EOD). Late onset depression is defined as late life depression with a first episode in older age and no history of depression in early age, with the common threshold being 60 [46] or 65 years old [47]. Early onset is late life depression with a more traditional course of onset, defined as having a first episode any time before older age, but typically early in life [46]. Importantly, as a subtype of LLD, EOD individuals experience continued depressive episodes into older age.

A foundational study in 1996 by Salloway et al. established late onset depression to be characterized by increased white matter hyperintensities compared to early onset depression [48]. This hyperintensity finding has been replicated many times [21,49], although both forms of LLD are characterized by a larger volume of white matter hyperintensities compared to younger cohorts with major depressive disorder [21]. Even after accounting for the effect of age, late onset individuals are more likely to have white matter abnormalities, encompassing both increased white matter hyperintensities [50] and reduced fractional anisotropy [51].

Both early and late onset depression are associated with cognitive impairments, but LOD has been shown to have worse cognitive impairment [52,53]. LOD is highly comorbid with vascular disorders and dementia [8] which has led to important etiological hypotheses for this LLD subtype. As LOD has been shown to have increased vascular risk compared to EOD [53], LOD has been posited to be etiologically related to vascular disease (particularly cerebrovascular disease) as early as 1997 [54]. Vascular depression is associated with white matter hyperintensities [55], although the underlying pathological process remains poorly understood. LOD’s association with dementia and Alzheimer’s Disease (AD) may also indicate an etiological relationship. An impactful study in 2002 [46] proposed depression to be a prodrome to AD, where early AD pathophysiology leads to neural degradation that causes depression, possibly before other AD specific symptoms arise [56]. However, evidence for a relationship between LLD and cognitive decline in the absence of amyloid pathology [57–60] appears to contradict the association between LOD and AD. This amyloid discrepancy may be explained by a recently developed concept entitled ‘suspected non-Alzheimer pathophysiology’ (SNAP) which refers to individuals without brain amyloid markers but with evidence for other abnormal markers of neurodegeneration [61–63].

Early onset late life depression on the other hand is not believed to have a different etiology than traditional understandings of Major Depressive Disorder. One particular feature of EOD is reduced hippocampal volume [64], which may relate to its cognitive impairment effect [65]. Hippocampal reduction is prominently found in late life depression but seems to be more specific to EOD as it is believed to be related to many lifetime episodes of depression [30,31]. However, the role of the hippocampus has been called into question with a 2017 meta-analysis which found more reduction in hippocampal volume in LOD as compared to EOD [25]. Given LOD’s relationship to dementia, more severe reduction in hippocampus volume in LOD would be etiologically consistent. In all, the hippocampus seems to be important in both EOD and LOD though the exact role is unclear. Other gray matter regions have been suggested to differentiate LOD and EOD, but there is very little consistency [66].

Table 1 presents a simplified overview of the differences between early onset LLD and late onset LLD. The indicators represent minimal change from non-depressed individuals (⊗), increase (↑), and decrease (↓), and the number of indicators represent the relative degree of increase/decrease.

## CHALLENGES AND FUTURE DIRECTIONS IN THE IDENTIFICATION OF LLD BIOMARKERS

### Inconsistent Findings

Although substantial progress has been made into identifying brain biomarkers of LLD, the convergence of findings across the literature has been limited. Systematic reviews into structural and functional brain correlates of LLD find that that even the most commonly reported brain correlates reach significance in only about half of the studies in which they are included [22,66]. For example, even the very robust finding in the field of reduced hippocampal volume was only found in 7 of the 15 studies included in the Sexton et al. systematic review and meta-analysis [22]. This meta-analysis also found an overall effect of thalamus reduction and yet only 1 out of the 3 included studies actually showed a decrease in thalamus volume [22]. Often meta-analyses fail to find any significant overlap between identified brain regions [1,22]. In particular, Saberi et al. 2022 [1], a recent pre-registered meta-analysis failed to find any coordinate-based overlap between results from 26 independent studies on the brain basis on LLD [1]. Saberi et al. 2022 included multiple modalities with an emphasis on functional connectivity and voxel-based morphology. Coordinate-based investigation is more rigorous than traditional meta-analysis procedures that compare effect size results of studies, which can be biased by the inflated effect size of small studies. While previous meta-analyses investigating structural volumes have found some consistent effects [22,25], Saberi et al. is the only recent meta-analysis to include functional connectivity findings in LLD. Systematic reviews investigating the differential neuroimaging correlates of LOD versus EOD have also failed to converge on consistent findings [66,67]. For example, the role of hippocampal volume between LOD and EOD could not be determined due to inconsistent findings between studies investigated in both Schweitzer et al. 2001 [46] and Toenders et al. 2019 [66]. In summary, inconsistent findings have been reported in studies investigating correlates of LLD in general and studies differentiating LLD subtypes, and across both structural and functional neuroimaging modalities.

### Reasons for Inconsistent Finding

There are several potential reasons for the inconsistent replicability of LLD neurobiological markers. As discussed below, these potential reasons include inconsistency in the construct of LLD, the presence of symptom and biological heterogeneity in the LLD population, variations in experimental decisions and rigor, and challenges with personalized psychiatry (Table 2).

### Construct Variability

The construct of LLD would benefit from enhanced standardization. Firstly, the evaluation of depression is inconsistent. Some studies evaluate depressive symptoms [68], some use a self-report questionnaire to identify depressed individuals [69], and some undergo a structured clinical interview [1,22]. Importantly, these approaches may capture different ranges of depression severity. Even within one approach there is variability, for example structured clinical diagnosis is the most consistent of the options and yet even diagnosis of depression has a kappa score (between rater agreement) of 0.43 [70]. Beyond diagnostic variation, LLD studies can have a wide variety of age thresholds for the definition of late life ranging from 50 years old all the way up to 70 years old in some cases [1]. Furthermore, inclusion and exclusion criteria are highly variable, for example restrictions on medications and comorbidities differ from study to study [22]. It is unclear to what degree these construct variations may drive inconsistencies in LLD biomarker findings.

### Etiological Heterogeneity

Another reason for inconsistent results is heterogeneity in LLD [22,23,71]. Here, we define heterogeneity as variability due to ‘true’ etiological differences between patients, as opposed to construct or analytical variability leading to experimentally introduced differences between LLD samples.

The heterogeneity of LLD creates challenges for research into neuroimaging correlates of LLD. Heterogeneity impacts patient-control group comparisons, which represent one of the most common experimental designs for research into neuroimaging correlates of LLD. In a highly heterogeneous patient group, estimating patient-control differences can result in either a reduced or null signal due to the combination of subgroups with inconsistent effects (especially in high sample size studies), or can result in a spuriously strong signal due to overrepresentation of a subgroup (more likely in small sample sizes). This type of sampling bias, which occurs when some members of the LLD population are systematically more likely to be selected in a study sample, may also be driven by oversampling of community-dwelling versus inpatient individuals [72,73] and may particularly impact underrepresented communities [74]. Beyond patient-control group comparisons, regression-style analyses are still fitted using data from all patients and therefore also do not account for heterogeneity.

In attempt to address heterogeneity, the National Institutes of Health launched the Research Domain Criteria (RDoC) initiative to encourage the development of novel approaches to the classification of mental disorders based on objectively measurable biological markers (i.e., identifying ‘biotypes’). However, the definition of biotypes typically only subtypes based on a single modality (such as structural neuroimaging [75], proteomics [76], or genetics [77]). Importantly, it is likely that there does not exist a single set of LLD biotypes that offer meaningful separation across all sources of heterogeneity (including, but not limited to, clinical, neurobiological, and genetic sources). If such a single set of LLD biotypes existed, this would imply a one-to-one mapping between all sources of heterogeneity, such that the same LLD subgroups must consistently differ in all sources of heterogeneity. There is some evidence for cross-domain consistency such as genetic differences in biotypes derived from structural neuroimaging [75].

However, the lack of consistency across biotype studies may suggest that the relationships between different sources of LLD heterogeneity is more complex, such that biotypes may be nested (many-to-one mapping) or may fundamentally differ (many-to-many mapping) between different sources of heterogeneity. For example, Late onset depression is believed to have a distinct etiology to early onset. An individual can have depression related to vascular disease or as a prodrome to Alzheimer’s. [8] Vascular depression has a different etiology to Alzheimer-related depression which has a different etiology to non-comorbid related depression, though have the same clinical profile (late onset). This would be an example of a many-to-one, or nested, brain-symptom relationship. Although multimodal attempts to identify biotypes are feasible, model optimization may prove challenging, and the validation of results becomes increasingly challenging and circular as more and more information is included in the data-driven biotype definition. As such, the complexity of interactions among sources of LLD heterogeneity greatly complicates research aiming to address the challenge of depression heterogeneity.

### Personalized Psychiatry

Despite widespread acknowledgement for the need to adopt individualized techniques to study, diagnose, and treat mental health [78–80] (LLD encompassed), important challenges exist here too. Neuroimaging measures of brain structure and function primarily calculate summary measures based on existing atlases that parcellate the brain into a set of regions with fixed boundaries. However, these atlases are typically estimated from healthy young adults and therefore may not be suitable for clinical and/or lifespan samples. Furthermore, the presence of variation in functional boundaries across individuals is increasingly recognized [81], highlighting the need for individual-specific estimates of brain organization. LLD research has yet to implement this line of approach so far as the authors are aware.

### Experimental Rigor

Beyond variation in LLD construct and LLD heterogeneity, there are additional experimental factors that may contribute to inconsistencies in findings. In the search for neuroimaging biomarkers of LLD there are many different processing steps, parameter decisions, and modeling options to choose from. Recent studies in the general neuroimaging domain have started to reveal the impact of these analytical decisions on downstream results [82–84]. In addition to analytical decisions, recent work has highlighted the role of sampling variability as an explanation of inconsistencies in findings [85]. Sampling variability refers to the fact that statical findings will vary across different samples. Importantly, the extent of sampling variability (i.e., the range of observed statistical findings across different samples) is determined by the sample size [85]. Historically, sample sizes of neuroimaging studies in general (and accordingly in LLD neuroimaging studies) were relatively small, in part due to the cost of acquisition. For example, the 2013 Sexton et al. [22] meta-analysis investigated studies with sample sizes ranging from 10 to 226 depressed individuals (average 54) and the 2014 Wen et al. meta-analysis [23] investigated studies with depressed patient sample sizes ranging from 13 to 106 (average 34). More recent samples sizes have increased, for example, Wen et al. 2022 [75] included 501 depressed individuals. Importantly, publication bias (i.e., preferential publication of significant results over null results) likely exacerbates the challenge of inconsistent findings [86–88].

## RECOMMENDATIONS FOR FUTURE RESEARCH

### Enhance Construct Validity

Field wide agreement on constructs and the development of clinical measures with high interrater reliability would certainly help address the problems with constructs in LLD, however this is unlikely to occur in the short term. In absence of these changes however, there are opportunities to improve the reliability of existing clinical measures. For example, recent work has shown that repeating measures for a construct with poor reliability and utilizing the average leads to improved reliability [89]. Furthermore, composite or summary scores representing weighted averages across multiple measures are also more reliable in ways that have shown to improve prediction performance [90]. Therefore, averaging or combining multiple less reliable measures can result in a more reliable composite measure.

### Parse LLD Heterogeneity

There is substantial work investigating the potential of subtypes within LLD. There has been some recent work establishing subtypes based on literature [91] and some work investigating data-driven subtypes based on clinical data [92]. However, investigations into data-driven subtypes of LLD based on neurobiological data is still in its infancy. One important study to do so is Wen et al. [75] who used a clustering algorithm called HYDRA to identify two biotypes of LLD that differed on genetic, neurobiological, and clinical features. In addition to biotype research that identifies discrete subgroups, alternative heterogeneity approaches include dimensional studies that aim to identify principal axes of continuous variation [93–96]. More work investigating data-driven LLD biotypes and dimensions will hopefully explain some inconsistency in LLD findings and lead to robust neurobiological markers.

An important challenge for LLD heterogeneity is the need to develop an understanding of the interplay between diverse sources of heterogeneity. This type of understanding is important to develop multimodal biotype algorithms that can model shared (one-to-one), nested (many-to-one) and unique (many-to-many) subgroup boundaries across sources of heterogeneity. One recent study aiming to map the interplay between different sources of heterogeneity in depression isolated individuals with identical clinical profiles (parsing clinical heterogeneity) and then applied data-driven clustering within the clinically isolated groups to find neurobiologically distinct subgroups [97]. This approach could readily be extended to LLD. As LLD likely has many sources of heterogeneity (even within neuroimaging, many modalities are necessary to capture the variability in gray and white matter), future work to map links between diversity sources of LLD heterogeneity will be crucial to understanding LLD brain biomarkers.

### Personalized Psychiatry

Individualized analytical approaches, also sometimes referred to as personalized psychiatry, to investigating neurobiological markers of LLD would also contribute to addressing the challenges caused by heterogeneity. Individualized approaches would be investigating neuroimaging at an individual level as opposed to a group level (i.e., quantifying and analyzing the signal of each participant). New statistical tools have been developed that would allow for this kind of investigation in vastly different ways.

There are new efforts to represent brain connectivity on an individual level [98]. One example is PROFUMO, the PRObabilistic FUnctional MOde. PROFUMO is a brain parcellation algorithm to determine resting state functional modes probabilistically that estimates both group and subject variability in the spatial and temporal modes of MRI [99]. There are many other examples of individualized parcellations such as template ICA [100] and hierarchical brain parcellation [101]. Many of these approaches adopt a hierarchical framework that leverages the group data to ensure correspondence across individuals, whilst optimizing for individualized measures of brain organization. Importantly, such hierarchical models leverage rich group data (which benefits from many more data points) as priors for individual estimates to overcome the challenge of limited individual data. An alternative to hierarchical models is precision functional mapping [102] which requires many repeated scans of the same person to get a highly robust representation without the use of group data.

A complimentary way to investigate brain features at an individual level is to quantify that individual’s brain feature compared to a healthy control group. Entitled normative modeling, one gets an individual level quantification of how far from normal each person’s brain feature deviates from a normative comparison cohort. Although normative modeling cannot capture individualized functional boundaries, it does offer a clinically meaningful approach to assess the distance between an individual patient and a group of healthy controls. Notably, normative modeling could be combined with the individualized measures of brain organization described above. Normative modeling has been applied to depression before [103], but has not yet been applied to LLD to the knowledge of the authors.

Some important obstacles preventing the widespread use of individualized approaches to overcome are computational intensity and lack of robust individual data. These individual level calculations can be extremely computationally costly, especially at large scales. There is also a lack of in-depth individual level data in most large-scale data acquisitions.

### Increased Rigor

Along with larger sample sizes, open science practices such as replications, code-sharing, pre-registration, and data-sharing are all ways to improve the experimental rigor towards clarifying LLD brain biomarkers. Marek et al. 2022 [85] showed that neuroimaging studies need approximately 2000 samples to avoid sampling variability and achieve stable findings based on realistic effect sizes in neuroimaging of mental health. Now that data-sharing is becoming increasingly common (and often required by funding agencies), larger sample sizes may become more feasible by combining multiple datasets, which may also contribute to improved generalizability and a wider range of phenotypes that can be investigated. Consortia efforts such as ENIGMA [104] and HARMONY [105] are important examples of shared data resources. The LLD literature also has very few replications of studies even though replications are crucial for confidence in findings [106]. Not only can subject variability in highly heterogeneous populations lead to spurious results, low statistical power and software errors among other reasons can lead to spurious findings that do not replicate [106]. Code sharing is an increasingly common practice that allows for more thorough investigation of the study as hand and facilitates replication. Another increasingly common practice is pre-registration, in which authors submit their research plans to a platform such as the Open Science Framework (http://osf.io/prereg/) prior to starting their studies, which improves transparency and helps prevent only publishing positive results. Details on best practices for pre-registration can be found here [107]. Some recent work in LLD have engaged in this practice, for example the Saberi et al. meta-analysis [1].

In Table 2, examples provided are to help understand the recommendation and suggest possible practical applications. Nevertheless, we note that community consensus on the best option will be important.

## CONCLUSIONS

In all, the field has made substantial progress in identifying brain biomarkers for LLD. LLD has been linked to increased white matter hyperintensity, reduced hippocampal volume, and default mode network abnormality among others. However, meta-analyses have highlighted important inconsistencies in the current literature. These inconsistencies can be explained by key challenges with construct variability, etiological heterogeneity, experimental rigor, and personalized psychiatry. These challenges can be addressed through novel approaches. Future work may focus on improving construct validity through measure repetition and the use of composite scores, improving experimental rigor through increased sample sizes and pre-registration, parsing heterogeneity through biotype research and studies into the relationship between diverse sources of heterogeneity, and adopting individualized analytical approaches to allow for more consistent results.

## Figures and Tables

**Table 1.** Overview of LOD/EOD differences.

Phenotype	Early Onset Depression (with episodes into late life)	Late Onset Depression (episodes starting in late life)
Brain marker: White Matter hyperintensities	↑	↑↑
Etiological Risk: Vascular Depression	⊗	↑
Etiological Risk: Dementia	⊗	↑
Cognitive ability	↓	↓↓

**Table 2.** Overview of challenges and recommendations for future research.

Challenge	Recommendations
Construct variability - Inconsistency in the use of measurements and thresholds - Poor reliability of clinical measures	- Develop novel clinical measurements with improved reliability - Adopt consistent clinical measurements across studies (e.g., using HAMD in all studies) - Adopt standardized age thresholds for LLD across studies (e.g., using 65 in all studies) - Adopt averaged measurements obtained from repeated measures (e.g., averaging over 3 repeated instances of HAMD) - Adopt composite scores (e.g., data-driven factor analysis on HAMD items)
Etiological heterogeneity - Reduced/null results due to combining distinct subgroups - Spuriously strong results due to overrepresentation of a subgroup (sampling bias) - Complex interplay between sources of heterogeneity due to true etiological variability in multiple domains (symptoms, neurobiology, genetics)	- Utilize data-driven approaches to identify biotypes driven by objectively measured biological information (e.g., data driven clustering on multimodal neuroimaging and genetics) - Perform comparisons across biotype studies to assess the degree of consistencies of resulting biotypes (e.g., Hannon et al. 2023 [108]) - Perform studies that develop an understanding of the complex interplay between diverse sources of LLD heterogeneity (e.g., approaches similar to Hannon et al. 2022 [97]) - Develop novel multimodal biotype algorithms
Personalized psychiatry - Brain organization varies between individuals and is often overlooked in atlas-based measures	- Hierarchical models that capture individual variation in relation to group averages - Precision functional mapping using large amounts of data from an individual (e.g., Gordon et al. 2017 [102]) - Normative modeling to estimate individual-specific deviation compared to a normative group (e.g., Rutherford et al. 2022 [109])
Experimental rigor - Inconsistent findings from small sample sizes - Bias towards positive (but potentially unreliable) findings due to publication bias	- Larger sample sizes to improve power and avoid sampling variability - Data sharing to encourage combined datasets and replication effects (e.g., best practices here [110]) More replications to test the generalizability of findings - Code sharing to enable replication and improve transparency (e.g., best practices here [110]) - Pre-registration to address publication bias and improve transparency (e.g., using the Open Science Framework)

## Data Availability

No data were generated from the study.
